# Non-pharmacological symptom self-management in non-malignant chronic disease: A scoping review

**DOI:** 10.4102/phcfm.v17i1.5095

**Published:** 2025-11-07

**Authors:** Lindsay Farrant, Helen Buchanan, Clare Ellis-Smith, Olivia Gaunt, Liz Gwyther, Richard Harding, Rene Krause, Alexandra Moors, Niri Naidoo, Sonwabiso Ngcowa, Kennedy Nkhoma, Jae Eun Park, Klaus von Pressentin, Matthew Maddocks

**Affiliations:** 1 Department of Family Community and Emergency Care, Faculty Health Sciences, University of Cape Town, Cape Town, South Africa; 2 Department of Health and Rehabilitation Sciences, Faculty of Health Sciences, University of Cape Town, Cape Town, South Africa; 3 Cicely Saunders Institute of Palliative Care Policy and Rehabilitation, Florence Nightingale Faculty of Nursing Midwifery and Palliative Care, King’s College London, London, United Kingdom

**Keywords:** symptoms, self-management, pain, dyspnoea, fatigue, non-pharmacological, chronic disease, palliative care, rehabilitation

## Abstract

**Background:**

Patients with advanced non-malignant diseases experience pain, dyspnoea and fatigue, requiring a rehabilitation approach within palliative care.

**Aim:**

To identify components of non-pharmacological interventions for symptom self-management for patients with non-malignant chronic disease.

**Method:**

This scoping review identifies: (1) systematic reviews of symptom self-management interventions for breathlessness, pain and fatigue in chronic lung, heart, renal and liver disease; (2) primary studies in low- and middle-income countries to identify intervention components, contextual factors, facilitators and barriers to symptom self-management. Six databases were searched, records exported to Rayyan and deduplicated. Following screening for inclusion, extraction was conducted. We conducted a narrative synthesis of intervention components and implementation factors, and content analysis of barriers and facilitators to interventions.

**Results:**

Thirty-one articles were included (21 systematic reviews and 10 primary studies). The populations studied had chronic lung disease (*n* = 19), heart disease (*n* = 12), chronic renal disease on dialysis (*n* = 2) and none had hepatic disease. The three most common intervention components were information, training and rehearsal for practical self-management activities and lifestyle support. Common patient barriers included motivation, adherence and health literacy, while facilitators encompassed knowledge, support and family involvement. The availability of healthcare workers can impact implementation, but remote access options should be considered.

**Conclusion:**

Disease and management information for patients and their family members, along with support for home application, form the foundation for effective symptom self-management.

**Contribution:**

Symptom self-management for non-malignant chronic diseases is uncommon in low-resource settings. This review outlines the necessary components and implementation considerations.

## Introduction

Palliative care aims to enhance the quality of life for patients and families experiencing serious health-related suffering.^1^ Nevertheless, the integration of palliative care into primary health care has been particularly slow in low- and middle-income countries (LMICs).^2^ This is especially concerning given the rising prevalence of non-communicable diseases (NCDs) within these regions, which are associated with reduced quality of life over the trajectory of chronic disease and increased use of emergency services because of uncontrolled symptoms.^3,4,5^ Three-quarters of patients in need of palliative care globally live in LMICs; the majority of these have NCDs and less than 10% receive the palliative care they need.^6^ A significant challenge in LMICs is the limited availability of palliative care that is integrated across various healthcare disciplines. Alongside clinical care and support, there is a need and opportunity to support patient self-efficacy^7^ and to reduce reliance on the healthcare system, particularly emergency care, for symptom management. Palliative rehabilitation encompasses interventions aimed at managing symptoms, enhancing comfort and promoting independence for as long as possible.^1^ Reviews of the evidence for self-management interventions have shown their benefit in improving self-efficacy, health-related quality of life, depression and anxiety for patients with chronic kidney disease,^8^ and in improving dyspnoea, health-related quality of life and reducing hospital admissions in patients with chronic obstructive pulmonary disease (COPD).^9^ Although rehabilitation services are inherently aligned with the objectives of palliative care, there is a notable lack of integrated practice between these fields in LMICs, and particularly limited access to these professional services for symptom self-management at the primary care and community level.^5^ This gap highlights an urgent need to identify effective rehabilitation strategies which can be employed within palliative care provision to improve the quality of life for patients with non-communicable illnesses.

This approach necessitates flexibility and the continual adaptation of care goals in response to changes in a patient’s condition. Traditionally, palliative rehabilitation is delivered by qualified professionals such as occupational therapists, physiotherapists, speech and language therapists, and dietitians. Common symptoms managed within palliative rehabilitation include fatigue, dyspnoea and pain. However, in LMICs, these skilled professionals are scarce, particularly at the community level. As a result, alternative models of delivery must be developed to ensure that patients and their families have access to rehabilitation that supports symptom management. A definition of symptom self-management interventions is provided by Jonkman et al.:^10^

Self-management interventions aim to equip patients with skills to actively participate and take responsibility in the management of their chronic condition in order to function optimally through at least knowledge acquisition and a combination of at least two of the following: stimulation of independent sign/symptom monitoring, medication management, enhancing problem-solving and decision-making skills for medical treatment management, and changing their physical activity, dietary, and/or smoking behaviour. (p. 35)

This scoping review is part of a wider project to develop and investigate the acceptability and feasibility of symptom self-management intervention for pain, breathlessness and fatigue for people living with chronic organ failure in South Africa. As part of the intervention development, this review aimed to identify the active components of non-pharmacological interventions for symptom self-management for patients with advanced non-malignant chronic lung, heart, renal and liver disease, specifically for the symptoms of breathlessness, pain and fatigue. Additional objectives included identifying the barriers to and facilitators of symptom self-management, and the contextual factors influencing symptom self-management in LMICs.

## Methods

A scoping review utilising two searches was conducted according to guidance by Peters et al.^11^ The first search identified systematic reviews which document the active components of interventions to support symptom self-management for the symptoms of breathlessness, pain and fatigue in advanced chronic lung, heart, renal and liver disease. The second search identified primary studies conducted in LMICs in order to identify specific contextual facilitators and barriers to symptom self-management in LMICs. We defined the term ‘advanced non-malignant chronic disease’ as referring to advanced disease which is deteriorating despite the best available medical management.^12,13,14,15,16,17,18,19,20^ Eligibility criteria are detailed in Table 1.

**TABLE 1:** Eligibility criteria.

Search	Inclusion criteria	Exclusion criteria
Search 1	Systematic reviews Self-management of symptoms of pain, breathlessness and fatigue Patients with chronic non-malignant renal, hepatic, respiratory and cardiac disease Conducted in HIC or LMIC settings Non-pharmacological interventions alone or in combination with pharmacological interventions English articles only, because of time and resource constraints Adult population Published since the year 2000	Studies other than systematic reviews Reviews that only included pharmacological interventions Reviews of populations other than chronic renal, hepatic, respiratory and cardiac disease or mixed populations where the findings are not disaggregated Reviews including symptoms other than pain, breathlessness and fatigue Reviews which did not disaggregate findings for pain, shortness of breath and fatigue from other symptoms Studies published before the year 2000
Search 2	Studies investigating self-management of symptoms for patients with chronic non-malignant renal, hepatic, respiratory and cardiac disease Studies investigating self-management of symptoms of pain, breathlessness and fatigue Studies conducted in LMIC settings^21^ Studies that include non-pharmacological interventions alone or in combination with pharmacological interventions Interventional studies (with or without contextual description or process evaluation) English language articles only, because of time and resource constraints Studies with an adult population Studies published from 2000 onwards	Studies published before the year 2000 Studies that only included pharmacological interventions Studies on populations other than chronic non-malignant renal, hepatic, respiratory and cardiac disease or mixed populations where the findings are not disaggregated Studies on symptoms other than pain, breathlessness and fatigue or which did not disaggregate findings for pain, shortness of breath and fatigue from other symptoms

Note: Please see the full reference list of the article Farrant L, Buchanan H, Ellis-Smith C, et al. Non-pharmacological symptom self-management in non-malignant chronic disease: A scoping review. Afr J Prm Health Care Fam Med. 2025;17(1), a5095. https://doi.org/10.4102/phcfm.v17i1.5095, for more information

LMIC, low- and middle-income countries; HIC, high-income countries.

### Search strategy and study selection

The search strategies were informed by methodological guidance on scoping reviews^11^ and drew on our previous review of symptom self-management interventions for people living with human immunodeficiency virus (HIV).^22^ For both searches, we used the following databases: Medline via PubMed; EBSCOhost for Academic Search Premier, Africa-Wide Information, CINAHL, ERIC, Health Source: Nursing/Academic Edition, APA PsycArticles, APA PsycInfo and SocINDEX; Scopus; Web of Science: Core Collection and SciELO only; and the Cochrane Library of Systematic Reviews, and Cochrane Register of Controlled Trials (CENTRAL). The search terms were guided by the scoping review concept of inclusion based on population, concept and context (PCC).^23^ Search one was conducted in February 2023 and updated on 04 November 2024. Search terms included (1) terms relating to chronic disease or end-stage disease or specifications for cardiac, hepatic, respiratory and renal disease; (2) terms relating to self-management and (3) terms relating to symptoms. Search two was conducted in June 2023 and updated on 11 November 2024. These search terms included (1) and (2) from search one and terms relating to LMICs. The groups of terms for both searches were combined using the Boolean Operator AND. For both searches, medical subject headings and keyword terms were used for exploring synonyms and a record was kept of all search terms and the results of all searches. The search strings for both searches that were used for PubMed are included in Online Appendix 1.

### Screening and study eligibility selection

All references were exported to EndNote. Once all database searches were complete, references were exported to Rayyan^24^ and duplicates were removed. Inclusion and exclusion selection were piloted by N.N., S.N. and L.F. with a sample of references to guide the subsequent screening process of titles and abstracts and full texts. A double-blind review was performed for half of the screened articles. Disagreements were discussed, and where uncertainty remained, N.N., H.B. and M.M. provided a third reviewer decision. The screening and selection process was captured on a Preferred Reporting Items for Systematic reviews and Meta-Analyses (PRISMA) flow chart.^25^

### Data extraction

Extraction for search one and search two used the same template. Contextual implementation factors were extracted for only the LMIC primary studies, using the first three domains of the (updated) Consolidated Framework for Implementation Research (CFIR).^26,27^ The data extraction template was reviewed ahead of extraction. Extraction was piloted by S.N., L.F., K.v.P., N.N. and H.B. The extraction spreadsheet was refined, and double-blind extraction was conducted. The extraction process included the following elements: first author, year, country, study setting, population, intervention descriptors (including intervention components, providers, number and duration of contact sessions, and duration of the intervention programme), identified implementation barriers and facilitators, and contextual factors. In cases where systematic reviews included studies with populations or symptoms not covered in this review, only the relevant disaggregated study populations and symptoms were extracted.

### Data synthesis

Narrative synthesis of the data considered intervention descriptors, intervention components, outcome measures and contextual factors. Intervention components were assessed and classified according to the Practical Reviews In Self-Management Support (PRISMS) taxonomy of self-management support^28^ (Table 2), each by two of four independent researchers (H.B., L.F., O.G. and M.M.). Content analysis of the factors relating to the barriers and facilitators of symptom self-management was conducted by two reviewers (H.B. and L.F.), categorising these to guide descriptive understanding.^29^ Narrative synthesis was conducted for the CFIR outer and inner domains.^26,27^ Outer setting considerations include patient needs, available resources and the extent of effective organisational linkages, while the inner setting refers to the structure, culture and communication within organisations leading the intervention.^30^

**TABLE 2:** Practical reviews in self-management support taxonomy of symptom self-management.

Component	Description
A1	Education or information about the condition and/or its management
A2	Information about available resources
A3	Provision of or agreement on specific action plans and/or rescue medication
A4	Regular clinical review
A5	Monitoring of the condition with feedback to the patient
A6	Practical support with adherence (medication or behavioural)
A7	Provision of equipment
A8	Access to support when needed
A9	Training and/or rehearsal to communicate with healthcare professionals
A10	Training and/or rehearsal for everyday activities
A11	Training and/or rehearsal for practical self-management activities
A12	Training and/or rehearsal for psychological strategies
A13	Social support
A14	Lifestyle advice and support

Note: Adapted from Pearce G, Parke HL, Pinnock H, et al. The PRISMS taxonomy of self-management support: Derivation of a novel taxonomy and initial testing of its utility. J Health Serv Res Policy. 2016;21(2):73–82. https://doi.org/10.1177/1355819615602725

### Ethical considerations

Ethical clearance to conduct this study was obtained during the intervention development phase of the Global Health and Palliative Care (GHAP) project at the University of Cape Town, Faculty of Health Sciences Human Research Ethics Committee (HREC REF: 317/2023). The protocol was prospectively registered with the Open Science Framework (OSF) Registries (https://doi.org/10.17605/OSF.IO/N4R87).

## Results

**FIGURE 1:**
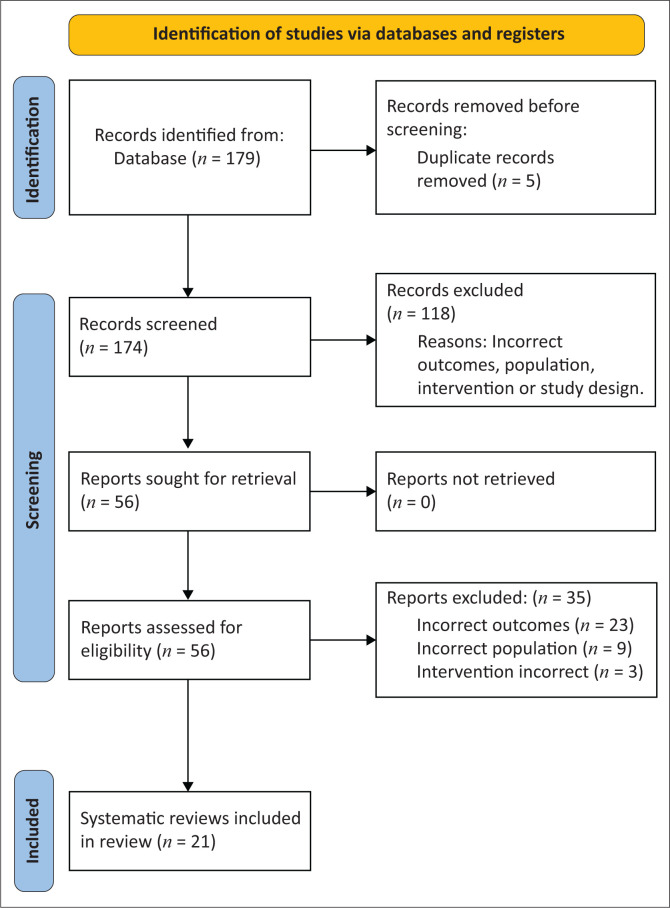
Preferred Reporting Items for Systematic Reviews and Meta-Analyses flow chart for search one.

**FIGURE 2:**
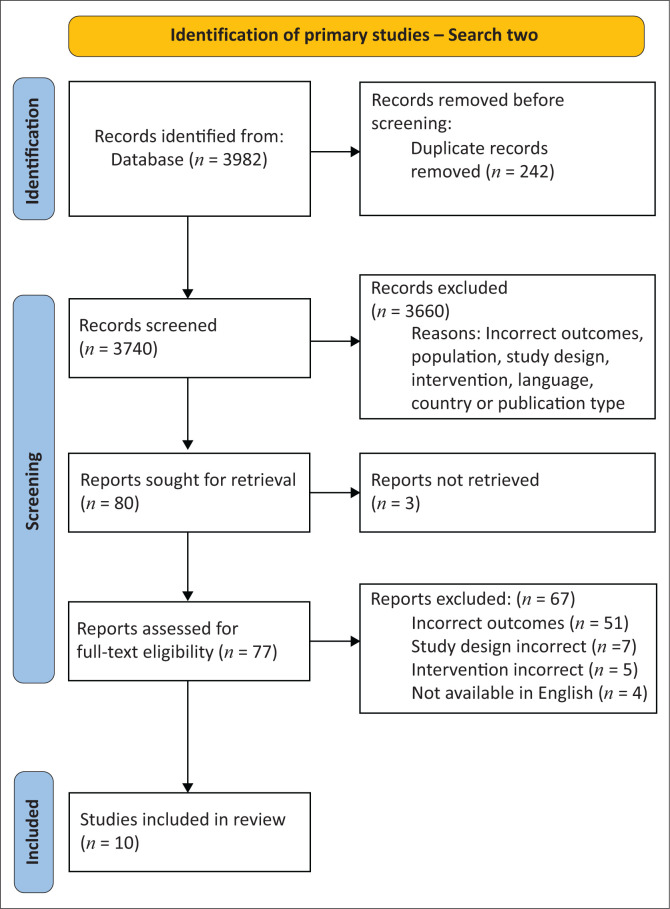
Preferred Reporting Items for Systematic Reviews and Meta-Analyses flow chart for search two.

In total, 31 articles were included across the two searches. Search one identified 174 articles after deduplication, and 21 systematic reviews met the eligibility criteria (Figure 1). Search two (Figure 2) yielded 3740 articles after deduplication, with 10 primary studies meeting the eligibility criteria. Figure 1 presents the PRISMA flow chart for search one and Figure 2 for search two.

### Characteristics of included articles

Six systematic reviews included relevant studies conducted only in high-income countries (HIC)^31,32,33,34,35,36^; four systematic reviews did not state the countries of included studies^9,37,38,39^ and 11 systematic reviews included relevant studies extracted from HICs and LMICs. The LMICs represented in the systematic reviews were Brazil,^40,41,42^ China,^40,42,43,44,45,46,47^ India,^42,48^ Indonesia,^42^ Iran,^41,46,49^ Thailand^49^ and Turkey.^41^ The included primary studies were conducted in China,^50,51,52^ Colombia,^53^ India,^54^ Iran,^55,56^ Nepal,^57^ Thailand^58^ and Turkey.^59^ No sub-Saharan African countries are represented by relevant studies in the included articles.

Included systematic reviews and primary studies will be presented together as a combined dataset, as ‘included articles’, unless otherwise specified. Most articles reported face-to-face (individual or group) interventions, while many interventions included face-to-face with a combination of telephonic and computer-based online interventions. Home visits, video-conferencing and a mobile app were less common. Most articles included participants with COPD or chronic lung disease (CLD) (*n* = 19), while just over half included participants with chronic heart disease (*n* = 12) and two articles included participants with chronic kidney disease on haemodialysis. No articles were included with interventions for patients with chronic liver disease. The duration of intervention varied, with the shortest being 6 days, the longest 48 months and an average duration of 10 weeks to 12 weeks. Interventions were delivered mainly by nurses and researchers, while some involved occupational therapists and physiotherapists, and some included a multi-professional team of health professionals. Some interventions included lay leaders in delivery. The number and length of sessions varied across studies, tailored to patient needs. The number, duration and setting of contact sessions varied widely. Table 3 identifies the countries and the settings in which the interventions were conducted, the intervention providers and study population recipients, the intervention duration, delivery modes, session number and session duration per participant.

**TABLE 3:** Characteristics of articles.

Author and Countries	Delivery mode	Providers	Recipients	Duration of intervention	Number and duration of contact sessions
**Systematic reviews of interventions in HICs and LMICs**					
Berry^31^ United States (US), Canada and Sweden	Face-to-face, telephone, computer-based component	Research assistant, cardiac nurse, nurse with group therapy expertise, research nurse, trained nurses	Chronic heart disease/CAD patients and spouses	7 weeks to 12 weeks	Overall range: 3–12 dyadic sessions Time per session: 20–60 min Telephone calls: 6
Buck^49^ US, Sweden, Thailand, New Zealand and Iran	Face-to-face, telephone and internet-based components	Not stated	Dyads consisting of a patient with heart failure and at least one informal caregiver	4 weeks to 12 months, where reported	Overall range: 1 face-to-face to 52 weekly interactive voice response call contacts Individual sessions: 1–3 Group sessions: 1–2 Home visits: 1 to deliver and train on equipment Telephone: 1–9; or in response to data Interactive voice response calls: 6–52
De Jong et al.^32^ US	Internet-based asynchronous communication; or face-to-face and telephone	Not stated	People with COPD or CHF	52 weeks	Not stated
Helvaci and Metin^44^ the Netherlands, United Kingdom (UK), China, Korea, Australia, Iceland and the US	Face-to-face, telephone and tele-monitoring; individual or group sessions	Nurses	COPD patients	2 months to 24 months	Overall range of at least 1–16 sessions Time: 15–45 min Home visits: 8 Phone calls: 2–16; up to 20 mins to 30 mins each reported; first session telephone coaching 35–60 min
Kwekkeboom and Bratzke^40^ US, China and Brazil	Face-to-face and telephone	Nurses	HF patients	6 days to 15 weeks	Overall range 2–15 sessions with 27 refresher sessions Time for sessions: 20 mins for inpatients to 45–90 min for other sessions Telephone calls: 3 for 10–15 min Exercise time: 20–30 mins daily to 15–30 min twice daily
Zwerink^9^ Not stated	Face-to-face, internet-based and telephone	Nurses	COPD patients	3 months to 24 months	Overall sessions: Range 1–40 Individual sessions: 1–32 (followed by 20 monthly physiotherapy sessions) to 112 Group sessions: 1–44 Home visits/sessions: 0–1 to 10 Range of session time: 20 min to 2 h Telephone calls: range of 2 in total to once per month for 22 months; Range of 10–20 min reported per telephone call Exercise time: From not mandatory to at least 3 times per week for 20–30 min; to daily 20–60 min
McGillion^37^ Not stated	Not stated; Individual and small group sessions	Not stated	Chronic stable angina patients	3 weeks to 8 weeks	Overall session range: 6–14 sessions If duration of sessions stated: 1 h per session
Mulligan^33^ Ireland, England and the Netherlands	Not clearly stated, appears F2F, stated telephone support calls. Individual and group sessions	Health professionals: OT, physiotherapists; pharmacists; dieticians; nurse; medical doctors. Lay leaders skilled in stroke and trained by the National Stroke Foundation. Coach-led (registered dietician and contracted fitness instructor) or self-directed via DVD	Lung or heart disease patients	6 weeks to 14 weeks	Number of sessions: 6–16 Duration of sessions: 2 h single session to 1–3 h per week Support phone call: 1 Individual motivational counselling: 1 h Exercise: 1 h, monthly drop in sessions
Qian, 2019^60^ Not stated	Not stated	Not stated	Advanced COPD patients	Not stated	Not stated Fan therapy duration: 5 min or not stated
Schadewaldt^45^ Australia, Canada, China and the UK	F2F and telephone, Individual or group	Nurses, trained in counselling techniques. Specialist cardiac liaison nurse	People with CHD	3 months to 18 months, to 10 years	Sessions range: monthly for 1 year Session duration: 1 h Telephone contacts: monthly for 1 year (10–15 min).
Sokalski^46^ UK, China, the US, Iran and Canada	F2F, home visit, telephone calls	Registered nurses trained in MI	People with HF	2 months to 5 months	Number of sessions: 3–8 Session duration: 15–20 min to 90 min Telephone call number: 3–12 Telephone duration: 5 min or not reported
Wakefield^34^ US, Europe and Australia	F2F; clinic or MD office visits; home visits; unscheduled telephone availability of staff; remote videophone; remote vital sign monitoring.	Nurses	Chronic heart failure patients	10 days to 20 months	Not stated
Wang^48^ India, the UK, Spain, Iceland, China, the Netherlands, Australia, the US, Sweden, Belgium, New Zealand and Canada	F2F, home visits, telephone calls	MDT = Clinical pharmacist; Physiotherapists; Physicians, GP, and visits to pulmonary physicians. Medical and nursing staff. Respiratory therapist. Nurse/respiratory educator	COPD patients	2 weeks to 48 months	Number of sessions: 2 to 4–96 Session duration: 30–45 min to 2 h initial SME training and weekly 1 to 3 h sessions for 7–8 weeks Structured telephone support: 2–6 calls Lectures: 48 in total, 40–60 min each Home care visits were offered: Individually tailored, at least two
Zhao,^41^ Turkey, Brazil, Australia, the US, Iran, Canada, Taiwan and the Netherlands	Not stated	Not stated	Participants with end-stage renal disease	2–12 months	Exercise session: Twice to three times per week for exercise; daily for muscle relaxation Exercise session duration: 15–90 min
Inouye^47^ China, England, New Zealand	Telephone, or not stated	Nursing staff, dieticians, diabetic educators, traditional Chinese practitioners and physical therapists	People with a CV or respiratory disease	1 months to 24 months	Session number: 3–29 Session duration: 1–3 h Telephone follow-up: Total of 2 to every two weeks until the 14th week Telephone call duration: 10–20 min Exercise practice at home: Twice a day or not stated
Ngai^35^ Hong Kong, Australia and the US	F2F (supervised) and remote (home sessions)	Qualified TCQ (*Tai Chi Qigong)* master; Physiotherapist who was an accredited trainer; physical education teacher; certified/professional Tai Chi master	COPD patients	6 weeks to 12 months	Tai Chi sessions: 5–7 days per week Session duration: 15–60 min Monitoring: Every 3 weeks, where reported
He^61^ North Africa, the US, Spain, Australia, The Nordic and Sweden	Not stated	Not stated	Individuals with severe/very severe COPD	6 weeks to 24 weeks	2 to 3 per week
Gurbuz and Demirel^36^ Canada, Sweden, Norway, the Netherlands and the US	Not stated	Not stated	Individuals with COPD	1 day to 8 weeks	1 to 24 F2F sessions
Bondarenko^39^ Not stated	Supervised, unsupervised, telephone follow-up, home visits and remote supervision via videoconference	Not stated	Individuals with chronic respiratory diseases	6 weeks to 2 years	Ranging from 10 to 120 min
Zhu^42^ China, India, Taiwan, Japan, Saudi Arabia, Indonesia, England, Germany, the Netherlands, Norway, Spain, Brazil, the US, Canada and Australia	Not stated	Not stated	Individuals with COPD	8 weeks to 3 years	2–7 sessions per week Each session ranged from 20 to 90 min
Chung^43^ Romania, the UK, Belgium, China, the Republic of Korea, Switzerland, the Netherlands and Taiwan	Mobile app-based vs centre-based care	Not stated	Individuals with COPD	3 weeks to 1 year	Not reported
**Primary studies conducted in LMICs**					
Wongpiriyayothar^58^ Thailand	F2F, at home and telephonic	Researchers	Patients with chronic heart failure	12 weeks	Total 3 Two home visits and at least two weekly telephone calls. 2 hours first session
Akyil^59^ Turkey	F2F individual	Researchers	COPD patients	3 months	4 contact sessions. Duration not stated.
Dehkordi^56^ Iran	F2F and telephone	Researcher	COPD patients	3–4 months	4 educational sessions once per week; 8 telephone calls
Nese^54^ India	F2F	Nurses	Patients with moderate or advanced COPD according to the GOLD criteria	10 weeks	1) 50 min training per patient. Daily application at home. 2) Average 20–25 min per patient. 10-min application 3 times a day at home.
Polkey^50^ China	F2F; after 12 weeks: at home or community group	Instructor and experienced physiotherapist supervisor	COPD patients	12 weeks	5 days per week for 1 h
Alishahi^55^ Iran	Smartphone application	Researchers	Haemodialysis patients	8 weeks	30 min every day
Sharma^57^ Nepal	Lectures, video clip display, group discussion, demonstration and individual counselling	Experts	COPD patients	6 weeks	2.5 h weekly
Tonguino Rosero^53^ Colombia	F2F programme, educational telephone call follow-up	Specialist physio in CPR	COPD patients	8 weeks	60 min, 3x per week 10–15 min education calls
Wen^51^ China	F2F and online. Telephone call follow-up	Not stated	CHD patients	12 week	One session bi-weekly, ranging 40–90 min
Fan^52^ China	Not stated	Researchers	COPD patients	Not stated	Not stated

Note: Please see the full reference list of the article Farrant L, Buchanan H, Ellis-Smith C, et al. Non-pharmacological symptom self-management in non-malignant chronic disease: A scoping review. Afr J Prm Health Care Fam Med. 2025;17(1):a5095. https://doi.org/10.4102/phcfm.v17i1.5095, for more information

COPD, chronic obstructive pulmonary disease; CHF, congestive heart failure; HF, heart failure; F2F, face-to-face; OT, occupational therapist; DVD; digital video disc; CHD, chronic heart disease; MI, myocardial infarction; MD, medical doctor; MDT, multidisciplinary team; CV, cardiovascular; GP, general practitioner; GOLD, global initiative for chronic obstructive lung disease; CAD, coronary artery disease; LMIC, low- and middle-income countries; HIC, high income countries.

### Intervention components

**FIGURE 3:**
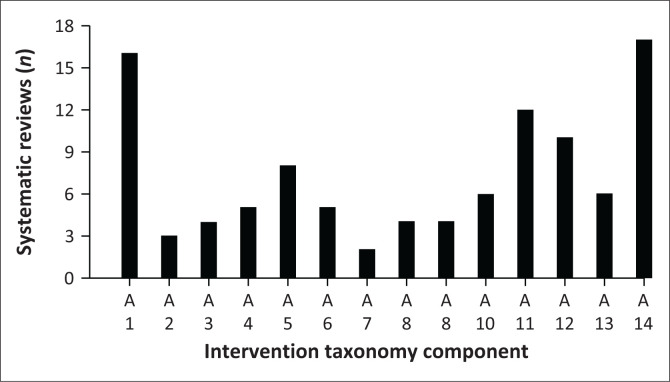
Number of systematic reviews that include individual PRISMS taxonomy components from search one. The x-axis represents PRISMS self-management taxonomy components A1–A14.

The reported intervention components are classified according to the PRISMS taxonomy of self-management support.^28^ All 14 of the PRISMS taxonomy components were reported by the included systematic reviews, while nine were reported in the included LMIC primary studies; therefore, the intervention components are reported separately. Of the 21 systematic reviews obtained in search one, the most common direct intervention components included were lifestyle and advice and support (A14) (*n* = 17/21, 81%), information about condition and/or its management (A1) (*n* = 16/21, 76%), training and/or rehearsal to communicate with healthcare professionals (A11) (*n* = 12/21, 57%), training/rehearsal for practical self-management activities (A12) (*n* = 10/21, 47%) and practical support with adherence (A5) (*n* = 8/21, 48%). The least common direct intervention components were the provision of equipment (A7) (*n* = 2/21, 9%) and information about available resources (A2) (*n* = 3/21, 14%), with the rest of the components being used in the interventions at an average of 23% (*n* = 5/21). Figure 3 details the number of reviews that included each component of the PRISMS taxonomy.^28^

**FIGURE 4:**
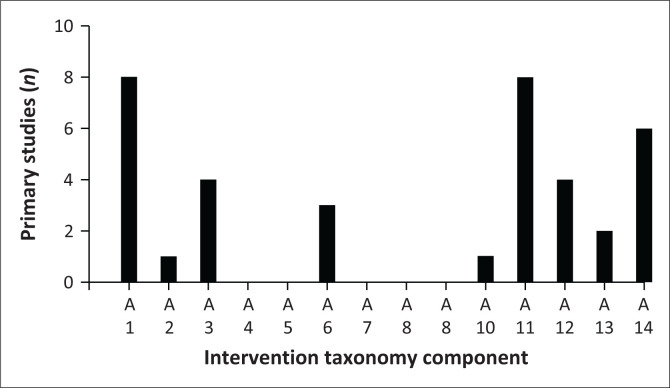
Number of primary studies that include individual PRISMS taxonomy components for search two. The *x*-axis represents PRISMS self-management taxonomy components A1–A14.

Figure 4 details the number of primary studies including PRISMS taxonomy^28^ intervention components as part of search two. Of the 10 primary studies included, the most common direct intervention components were training or rehearsal for practical self-management activities (A11) (*n* = 8/10, 80%), education/information about the condition and/or its management (A1) (*n* = 8/10, 80%) and lifestyle advice and support (A14) (*n* = 6/10, 60%). Compared to the systematic reviews, the LMIC primary studies included a narrower range of intervention components.

Table 4 reports information on the study setting, population and intervention components according to the PRISMS taxonomy. Online Appendix 2 reports details of the components of interventions for each included article.

**TABLE 4:** Study setting, population and intervention components.

Author	Setting	Disease population	Intervention components interpreted according to PRISMS taxonomy													
			A1	A2	A3	A4	A5	A6	A7	A8	A9	A10	A11	A12	A13	A14
**Systematic reviews of interventions in HICs and LMICs**																
Berry^31^	Treatment clinic, homes or hospital	CAD or CHF	+	-	-	-	-	+	-	+	-	-	-	+	+	+
Buck^49^	Hospital; clinical research centre; academic medical centres; outpatient clinic; home or primary care	HF	+	+	+	+	+	+	+	-	+	+	+	+	+	+
De Jong, Ros and Schrijvers^32^	Not stated	COPD or CHF	+	-	-	-	+	+	-	-	-	-	-	-	-	-
Helvaci and Metin^44^	Clinic or home	COPD	+	-	-	-	+	-	-	-	-	+	+	-	-	+
Kwekkeboom and Bratzke^40^	Home	HF	-	-	-	-	-	-	-	-	-	-	+	+	-	+
Zwerink^9^	Home; hospital; GP; outpatient clinic; primary care; university exercise facility; community; private physiotherapy practice or rehabilitation setting	COPD	+	-	+	+	-	-	-	+	-	-	+	+	-	+
McGillion^37^	Clinic, primary care practices or hospital	Heart disease	+	-	-	-	-	-	-	-	-	+	+	+	-	+
Mulligan^33^	GP; hospital; cardiac rehabilitation centre or cardiology outpatient clinics	COPD, CLD, CVD, CHD or CHF	+	-	+	-	-	-	-	-	-	-	+	+	-	+
Qian^38^	Hospitals; clinics or hospices	COPD	-	-	-	-	-	-	+	-	-	-	-	-	-	-
Schadewaldt^45^	Primary care; acute care hospital or tertiary medical centres; GP or home	CHD	+	-	-	+	-	-	-	-	-	-	+	-	+	+
Sokalski^46^	Community; hospital or heart failure clinic	HF	+	-	-	-	-	-	-	-	-	-	+	-	-	+
Wakefield^34^	Hospital; clinic; physician office or homes	HF	+	-	-	+	+	+	-	-	-	-	-	+	+	+
Wang^48^	Hospital; community or outpatient clinics; primary health care centres; lung physicians’ office or rural health clinic	COPD	+	+	+	-	-	+	-	+	-	+	+	+	-	+
Zhao^41^	Not stated	ESRD on haemodialysis	-	-	-	-	-	-	-	-	-	-	+	-	-	+
Inouye^47^	Home or clinic	HF, CVD, COPD or CLD	+	-	-	-	+	-	-	-	+	+	+	+	+	+
Ngai^35^	Hospital or clinic	COPD	-	-	-	-	+	-	-	-	-	-	+	+	-	-
He^61^	Not stated	COPD	+	-	-	-	+	-	-	-	-	-	-	-	-	+
Gurbuz and Demirel^36^	Supervised facility	COPD	-	-	-	-	-	-	-	-	-	-	-	-	-	+
Bondarenko^39^	Centre-based and home-based	CLD	+	-	-	-	-	-	-	-	+	-	-	-	-	+
Zhu^42^	Not stated	COPD	+	+	-	+	-	-	-	-	-	+	-	-	-	+
Chung^43^	Centre-based and home-based	COPD	+	-	-	+	+	-	-	+	+	-	-	-	+	-
**Primary studies conducted in LMICs**																
Wongpiriyayothar^58^	Home	CHF	+	-	+	-	-	-	-	-	-	-	+	-	-	+
Akyil^59^	Outpatient clinic at hospital	COPD	+	-	-	-	-	-	-	-	-	+	+	+	+	+
Dehkordi^56^	Hospital inpatient and home after discharge	COPD	+	-	+	-	-	?	-	-	-	-	+	-	-	+
Alishahi^55^	Home	CKD on haemodialysis	+	-	-	-	-	-	-	-	-	+	+	+	-	+
Nese^54^	Hospital outpatient clinic and home	COPD	-	-	-	-	-	-	-	-	-	-	+	-	-	-
Polkey^50^	Hospital	COPD	-	-	-	-	-	-	-	-	-	-	+	-	-	-
Sharma^57^	Respiratory and critical care unit	COPD	+	-	-	-	-	+	-	-	-	+	+	+	-	+
Tonguino Rosero^53^	Clinic	COPD	+	-	-	-	-	-	-	-	-	-	-	-	-	-
Wen^51^	Community health centres	CHD	+	-	-	+	-	-	-	-	-	-	+	-	+	+
Fan^52^	Community health centre	COPD	+	-	-	+	-	+	-	-	-	-	+	-	-	+

Note: Please see the full reference list of the article Farrant L, Buchanan H, Ellis-Smith C, et al. Non-pharmacological symptom self-management in non-malignant chronic disease: A scoping review. Afr J Prm Health Care Fam Med. 2025;17(1):a5095. https://doi.org/10.4102/phcfm.v17i1.5095, for more information

COPD, chronic obstructive pulmonary disease; CHF, congestive heart failure; HF, heart failure; CHD, chronic heart disease; MI, myocardial infarction; CV, cardiovascular; GP, general practitioner; CAD, coronary artery disease; ESRD, end stage renal disease; CLD, chronic lung disease; CKD, chronic kidney disease; LMIC, low- and middle-income countries; HIC, high income countries.

### Barriers

Eight studies^9,32,33,42,43,46,52^ reported barriers which were categorised^29^ into patient factors (seven barriers), health system factors (three barriers), health professional factors (two barriers) and an intervention factor (one barrier) (Table 5).

**TABLE 5:** Barriers to symptom self-management.

Factors	Barriers
Patient	Low adherence^42,43,46^ Use of technology^32^ Lower attendance rates for community-based intervention and rehabilitation programmes^33^ Limited health literacy^33,46,56^ Multiple co-morbidities and taking multiple medications^46^ Cognitive impairment^46^ Lack of (or low) motivation^43,56^ which can be linked to emotional factors and physical symptoms which perpetuate low motivation^56^
Health system	Lack of nutritional support to help patients maintain adequate BMI and increase their muscle mass^43^ Limited resources for in-person rehabilitation^43^ Limited availability of norms and standards for community healthcare and community nursing^52^
Health professional	Language barriers^9^ Low referral rates to rehabilitation programmes^33^
Intervention	Short study periods may limit efficacy and interpretations of findings^43^

Note: Please see the full reference list of the article Farrant L, Buchanan H, Ellis-Smith C, et al. Non-pharmacological symptom self-management in non-malignant chronic disease: A scoping review. Afr J Prm Health Care Fam Med. 2025;17(1):a5095. https://doi.org/10.4102/phcfm.v17i1.5095, for more information

BMI, body mass index.

### Facilitators

Five studies^32,42,43,46,55^ reported facilitators, which were categorised^29^ into patient factors (five facilitators) and intervention factors (four facilitators). Electronic messaging,^32^ information and education about the medical condition/symptoms^46^ and light exercise^42^ were identified as facilitators to effective self-management of the symptoms (Table 6).

**TABLE 6:** Facilitators of symptom self-management.

Factors	Facilitators
Patient	Knowledge about the disease process^46^ Support to address self-care barriers^46^ Family member/s involvement in care^46^ Patient motivation^46^ Asynchronous electronic messaging opportunities between patient and HCP may support subsequent shared decision-making^32^
Intervention	Telephonic contact and in-person interactions with participants increase implementation fidelity^55^ Acceptability to participants was increased with low-intensity exercise due to patient comfort, allowing longer participation in the intervention programme. An example was exercise in water, which offers buoyancy for low-impact resistance^42^ Mobile app-based rehabilitation may improve accessibility for patients with geographical and time limits^43^ Adjustable exercise regimes on a mobile app (to match participant exercise capacity)^43^

Note: Please see the full reference list of the article Farrant L, Buchanan H, Ellis-Smith C, et al. Non-pharmacological symptom self-management in non-malignant chronic disease: A scoping review. Afr J Prm Health Care Fam Med. 2025;17(1):a5095. https://doi.org/10.4102/phcfm.v17i1.5095, for more information

HCP, healthcare professionals.

Contextual implementation factors were not routinely considered or described in the primary studies, but when discussed, they were related to the reported barriers and facilitators. In the CFIR outer setting domain, design for local culture and setting^55^ was described, with consideration of healthcare professionals’ availability and lack of access to pulmonary rehabilitation.^59^ Patient outcome follow-up was reported as challenging because of geographical and telephonic access limitations.^56^ One research team conducted an assessment of available local resources alongside the assessment of how to apply national policies to support implementation.^51^ Another article made a recommendation for policy change where the need for care was not met by the current policy.^58^ Chronic medication costs borne by patients and their lack of time off work to attend primary care facilities made low-cost exercise self-management interventions an important consideration in LMICs.^50^

The CFIR inner domain^26,27^ highlighted the centrality of relational connections for symptom self-management implementation. Family involvement appears very important,^59^ in particular to support understanding, psychological coping and practical application of symptom self-management.^51^ Regular telephonic or in-person engagement from researchers or healthcare providers supports adherence to self-management,^55^ with role clarity and relationship development supporting this process.^57^ The availability of telephonic or application-based text communication was described as important for advice and support of patients in their application of self-management.^51,57^ Emotional coping support and social activity consideration were recommended as necessary for a recipient-centred intervention.^59^ The benefit of cost, time and space utilisation for symptom self-management designed for the home was clearly beneficial for patients in the community.^50,58^

## Discussion

This study details symptom self-management interventions for pain, shortness of breath and fatigue for patients with chronic lung, heart and kidney disease. The patient populations most commonly investigated in both searches were individuals with chronic lung diseases, specifically COPD, followed by chronic heart disease patients, where heart failure and coronary artery disease populations were most common. One systematic review^41^ and one primary LMIC study^55^ included interventions for individuals with chronic renal diseases with all undergoing haemodialysis at the time of intervention. These articles do not represent the large group of patients in LMICs who need but are not able to access dialysis for varying reasons.^62,63^

Because of the global increase in prevalence of chronic conditions, supporting self-management will increasingly become a core response of healthcare systems^28^ to improve patients’ quality of life.^64^ A tertiary systematic review of health service components for patients with advanced progressive chronic conditions showed that patient and family education and patient self-management are common components of effective services.^65^ Symptom self-management cannot be the responsibility of the person living with the chronic disease alone, but requires ongoing engagement between the patient, family caregivers, the healthcare professionals (HCPs) and the community-based care team.^28,32,46,55,66^ The PRISMS self-management taxonomy allows for the conceptualisation of self-management support and enables improved reporting of interventions and a clearer guide for the recommended components to optimise patient care.^28^ Interventions need not include all components, but consideration should be given to all the domains of self-management, and application should be contextualised and individualised as much as possible so as to tailor the intervention in a person-centred manner.^28^

The three most commonly included taxonomy components, A1 (information-giving), A11 (training/rehearsal for practical self-management activities) and A14 (lifestyle advice and support), are a low-resource way of supporting symptom self-management. The most common active PRISMS intervention taxonomy component^28^ identified was education (or information) about the condition and/or its management (A1). Education with the provision of information is a necessary starting point for any patient and family member to be able to increase their knowledge and understanding to start and then maintain self-management of their illness and their consequent symptoms.^67^ Information given, understood and retained by patients and family members is a part of the process of enabling self-determination^68^ and self-efficacy.^7^

Our findings show that information-giving and education can be approached in a variety of ways, all of which need consideration for relevance, acceptability and accessibility for the population and context concerned.^67^ This is supported by the findings of a systematic review of self-management interventions specifically conducted in primary care settings.^64^ Disease and management understanding also supports the patient’s understanding of their prognosis.^68^ A recent systematic review of interventions to improve patient prognostic understanding in advanced disease shows that such interventions that are specific for decision support, communication or a palliative approach appear to support appropriate prognostic understanding, but that this is a challenging outcome to achieve.^69^

Bayly et al.^65^ recommend that collaborative patient care that considers patients’ needs and not just diagnosis and management of acute events is supportive of effective care for patients with progressive disease. This should be considered in conjunction with a longer-term approach to supporting the implementation of symptom self-management that actively includes the patient and family and actively plans follow-up.^65^ Support for patients to learn and then develop skills in self-management can enhance self-efficacy.^64^ Repetition of information and assisting patients to understand how to embed self-management activities within day-to-day activities are necessary, and this underpins the importance of the need for supportive training and rehearsal of the taught self-management strategies.^28,64^ Healthcare professional roles in the process should include regular review^9,45,49^ and support for patient-directed goal setting^31,33,37^ with the collaborative action plans.^9,28,33,48,52,58,64^ The practical training and development of skills as part of symptom self-management requires active engagement from the healthcare provider.^33,48,51,56,58^

In terms of intervention implementation facilitators, there was no facilitator more common than another in either search. However, an underlying theme was that technology improved accessibility to the interventions and also adherence. Our findings show that patient adherence to self-management was noted as an important barrier. Understanding the challenges and reasons for limited adherence was less clear, although disease and management understanding are linked to adherence to medication,^23,34^ and self-care education has been shown to increase adherence to self-management.^70^ Patients need to feel motivated to engage and take responsibility for decision-making and their self-care.^71^ Motivational interviewing is a strategy in supporting self-efficacy towards effective symptom self-management,^33,46^ as are cognitive behavioural interventions.^47^ However, these formal approaches may be considered resource-intensive options, which may not be widely accessible in LMIC primary care settings. Further assessment is required of the access to resource-appropriate symptom self-management support for all those who require such support in primary care and community settings in LMICs. Electronic messaging, education on condition and light exercise were identified as facilitators to effective self-management of the symptoms. In the LMIC context, the latter two are practical and implementation may be set in motion through the healthcare workers, with additional training, development and support. Electronic messaging has been successfully applied,^32,44,49^ but resource constraints in LMICs mean that sustainability should be considered. Hearn et al.^72^ found that the use of phone calls and short message service (SMS) messages is effective for supporting self-management and does not require smartphones. However, the benefit of smartphones for playing video instructions was found to improve quality of life for patients with malignant disease in India,^73^ so smartphone use should not be discounted because of resource constraints.

Exercise is commonly applied as a symptom self-management intervention. While a range of exercises can be considered appropriate, single-limb exercises may not be sufficiently effective for shortness of breath or fatigue.^36^ Findings from our review suggest that exercise needs to be appropriate for cultural, contextual and resource considerations, as well as being individualised and person-centred, with ongoing support for implementation. The delivery of exercise interventions is acceptable when delivered by physiotherapists,^74^ but may be delivered by other professionals^35,48,52,55,58^ such as nurses.^34,44^

Relaxation and psychological strategies are recognised as important and necessary components of symptom self-management interventions, supporting patients and families to cope practically with psychological distress.^9,33,37,40,47,48,54,59^ Relaxation techniques are considered integral to the self-management of shortness of breath in chronic disease, and include use in crisis moments.^75,76^ Progressive muscle relaxation and deep breathing exercises appear to be effective in improving shortness of breath and fatigue in patients with COPD.^54^

The provision of information is an effective, low-resource and potentially time-efficient strategy, with the potential for patient benefit. Interventions requiring increased resources were less common, such as the provision of equipment (A7), access to support when needed (A8) and any intervention component with time-intensive rehearsal and training. In LMIC settings, the monitoring of the condition with feedback to the patient (A5) was not included at all. Monitoring the patient and providing feedback requires sufficient HCP time, which is a limited resource. Information about available resources (A2) and access to support when needed (A8) requires HCPs to have sufficient knowledge regarding local area resources and services. Gaining this knowledge requires understanding and time investments into ensuring provisions are adequate, as well as potential financial investments in resources to improve the standard of care. This is where the importance of collaborative approaches to self-management is highlighted. Multi-disciplinary healthcare teams and community care services together can facilitate appropriate coordinated care and enhanced self-management along the life course, particularly where formal rehabilitation services are scarce.^2,5,77^

### Limitations and methodological considerations

The 21 systematic reviews included in this review allow for the representation of a comprehensive review of the published literature on the active components included within symptom self-management interventions for pain, shortness of breath and fatigue in the study populations. This provides a robust contribution to the planning and development of non-pharmacological interventions for individuals with chronic diseases experiencing breathlessness, pain and fatigue. There are a number of methodological limitations to this scoping review. Not all records were screened by two independent reviewers. Data synthesis and categorisation by the PRISMS taxonomy was necessarily conducted based on the reported intervention descriptions, which were not always sufficiently detailed for certainty around the classification and therefore relied on interpretation. No studies were identified among populations with chronic liver disease or with advanced renal disease not receiving dialysis.

## Conclusion

Symptom self-management interventions play a significant role in reducing the prevalence and burden of physical symptoms in non-malignant conditions, improving patients’ feelings of self-efficacy and reducing dependence on health providers. This scoping review presents a practical descriptive overview of non-pharmacological symptom self-management for the common symptoms of pain, shortness of breath and fatigue as experienced by patients with non-malignant chronic respiratory, cardiac and renal disease. The categorisation of interventions according to the PRISMS taxonomy provides a classification of intervention options for researchers and clinicians. Accurate and understandable information is the basis for symptom self-management, but family involvement and support are also required to support rehearsal and application in the home. The synthesis of facilitators, barriers and LMIC setting implementation considerations highlights the importance of interventions being culturally and contextually relevant, as well as needing to be accessible for patients who experience functionally limiting symptoms in addition to geographical and financial access challenges. Interventions can be successfully applied by a diverse range of actors, including lay implementers such as health workers.
